# Effects of electrical stimulation on the cardiogenic differentiation of mesenchymal stem cells: A systematic review

**DOI:** 10.1007/s10616-026-01065-w

**Published:** 2026-10-17

**Authors:** Alejandra Sandoval Villarreal, Paola Neuta-Arciniegas, John Jairo Cabrera-López, Álvaro José Rojas Arciniegas

**Affiliations:** 1 Research Group INBIOMIC, School of Bacteriology and Clinical Laboratory, Universidad del Valle, Calle 4B # 36B-37, Cali, 760030 Colombia; 2 Faculty of Engineering and Basic Sciences, Universidad Autónoma de Occidente, Calle 25, Cali, #115-85, 760030 Colombia

**Keywords:** Mesenchymal stem cells, electrical stimulation, cardiogenic differentiation, cell viability, tissue engineering

## Abstract

Electrical stimulation has emerged as a promising strategy for promoting cardiogenic differentiation of mesenchymal stem cells; however, the available evidence remains highly heterogeneous across stimulation parameters, experimental platforms, and outcome measures. This systematic review synthesized the available in vitro evidence regarding the effects of electrical stimulation on MSC cardiogenic differentiation, with particular emphasis on stimulation parameters, experimental platforms and biological outcomes related to early cardiogenic commitment and structural maturation. A systematic search was conducted in seven electronic databases in accordance with the Preferred Reporting Items for Systematic Reviews and Meta-Analyses (PRISMA) 2020 guidelines. Fifteen preclinical in vitro studies met the eligibility criteria. Human-derived MSCs were the most frequently investigated cell source and carbon-based electrodes were the most commonly reported conductive material. Across the included studies, electrical stimulation was frequently associated with morphological changes and increased expression of markers related to early cardiogenic commitment and structural maturation, although findings were inconsistent for some outcomes. A stimulation frequency of 1 Hz, pulse widths of 5 ms, and stimulation periods of approximately two weeks were the most frequently employed conditions. Nevertheless, substantial heterogeneity in stimulation parameters, experimental platforms and outcome measures precluded direct comparisons among studies and prevented identification of an optimal stimulation protocol. Overall, the available evidence suggests that electrical stimulation may represent a promising strategy for promoting the cardiogenic differentiation of mesenchymal stem cells in vitro; however, greater methodological standardization, standardized outcome assessment and functional characterization are required before definitive conclusions regarding its effectiveness and optimal stimulation protocols can be established.

**Graphical abstract:**

**Figure:**
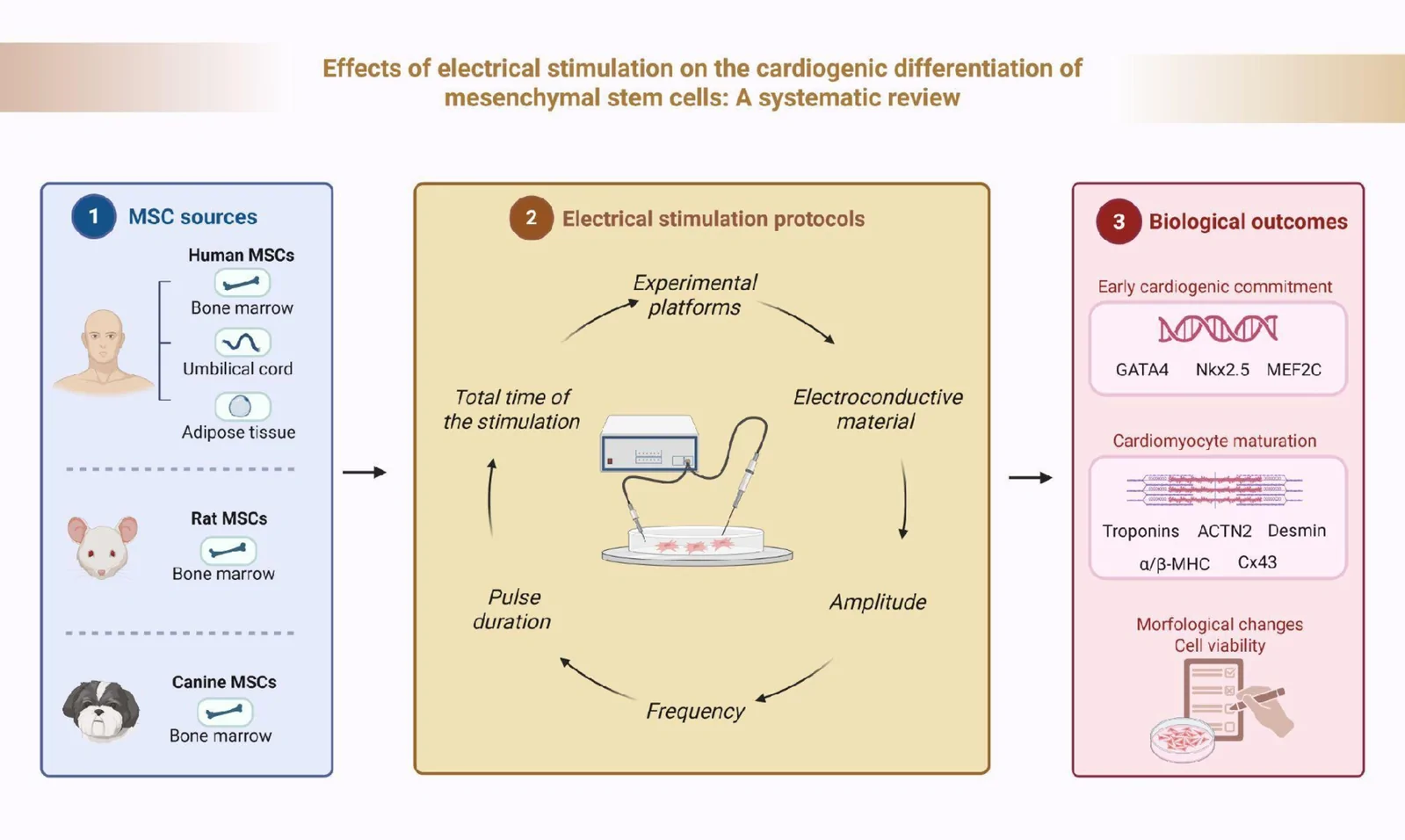

**Supplementary Information:**

The online version contains supplementary material available at https://doi.org/10.1007/s10616-026-01065-w.

## Introduction

Despite advances in medicine, cardiovascular diseases remain the leading cause of morbidity and mortality worldwide, with acute myocardial infarction representing one of the most prevalent and clinically significant manifestations (Redd et al. 2017). Because the adult myocardium has limited regenerative capacity, ischemic injury results in irreversible cardiomyocyte loss and replacement of the damaged tissue by fibrotic scar, ultimately impairing cardiac contractile function (Herreros et al. 2011). Consequently, regenerative strategies such as mesenchymal stem cell (MSC)-based therapy have attracted considerable interest for cardiac repair.

Among the different stem cell populations investigated for cardiac regeneration, MSCs remain one of the most extensively studied because of their low immunogenicity, immunomodulatory properties, ease of isolation and in vitro expansion, compatibility with tissue engineering approaches, and secretion of proangiogenic and antifibrotic factors that support myocardial repair (Abdi et al. 2008; Pittenger et al. 2004; Hare et al. 2012; Steens et al. 2018). In addition, compared with pluripotent stem cells, MSCs present a negligible risk of teratoma formation (Ben-David et al. 2011). They can be obtained from multiple adult tissues, making them promising candidates for further preclinical investigation in cardiac regenerative medicine. However, one of the main challenges limiting their therapeutic application is their low efficiency at differentiating into mature, functional cardiomyocytes. Although MSCs can express cardiac-specific markers under appropriate induction conditions, they generally retain their stromal phenotype and fail to achieve complete structural and functional maturation (Rose et al. 2007, 2008).

Limited cardiomyocyte maturation has been associated with poor electrical integration between transplanted MSCs and the host myocardium, increasing the risk of arrhythmias and reducing long-term therapeutic efficacy (Almeida et al. 2015; White et al. 2020; Soma et al. 2024). Furthermore, currently available strategies for inducing cardiogenic differentiation of MSCs rely on approaches such as treatment with demethylating agents, viral gene delivery, or prolonged coculture with cardiomyocytes (Shafy et al. 2009; Stoppel et al. 2016; White and Chong 2020). Although these approaches can promote the expression of cardiac markers, they remain limited by variable differentiation efficiency, poor reproducibility, and challenges in safety and clinical translation.

Consequently, considerable interest has emerged in electrical stimulation as an alternative strategy for modulating the cardiogenic differentiation of MSCs by mimicking key bioelectrical features of the native myocardial microenvironment (Dai et al. 2021; Eng et al. 2016; Yoshida et al. 2019; Tandon et al. 2009). Proposed mechanisms include modulation of intracellular calcium signaling, activation of calcium-dependent signaling pathways, regulation of cardiac transcription factors and gap junction proteins, cytoskeletal remodeling, and enhanced intercellular electrical coupling (Tandon et al.^2011^). Although several studies have reported encouraging findings, the available evidence remains methodologically heterogeneous across stimulation parameters, experimental platforms, MSC sources, and outcome measures. This variability has limited direct comparisons among studies. It has hindered the identification of reproducible stimulation strategies and the interpretation of the relative contribution of individual stimulation parameters to cardiogenic differentiation, cell viability, and cardiomyocyte maturation.

To address these challenges, this systematic review aimed to synthesize evidence from preclinical in vitro studies evaluating the effects of electrical stimulation on the cardiogenic differentiation of mesenchymal stem cells, with a focus on stimulation parameters, experimental platforms, and outcomes related to early cardiogenic commitment and cardiomyocyte maturation. In addition, this review sought to identify methodological trends, limitations, and knowledge gaps to inform future research and support the standardization of electrical stimulation strategies for cardiogenic differentiation of MSCs.

## Methodology

### Search strategy

The review question was formulated using the PICO framework: Which electrical stimulation strategies (parameters, configuration, and experimental platform design) have been used to promote cardiogenic differentiation of mesenchymal stem cells in vitro, and what evidence has been reported regarding early cardiogenic commitment and cardiomyocyte maturation?

The primary outcome was the effect of electrical stimulation on cardiogenic differentiation, assessed by the expression of markers of early cardiogenic commitment, cardiomyocyte maturation, or acquisition of a cardiac phenotype. Additional information on cell viability, morphological characteristics, electrical stimulation parameters, and experimental platform characteristics was extracted to characterize the available evidence comprehensively.

A systematic search was conducted in seven databases (PubMed, Ovid, EBSCO, Web of Science, Scopus, Virtual Health Library [BVS], and ProQuest) using controlled vocabulary and free-text terms related to mesenchymal stem cells, electrical stimulation, and cardiomyocytes. Searches were last updated on July 9, 2026. Depending on the database, search terms were applied to title and abstract fields or expanded to all searchable fields where appropriate. Studies published in English, Portuguese, Spanish, French, or Mandarin during the previous 20 years were considered eligible. The complete search strategies for all databases are provided in Online Resource 1.

Eligible studies were published preclinical in vitro investigations evaluating the effects of electrical stimulation on the cardiogenic differentiation of mesenchymal stem cells. Cardiogenic differentiation was assessed by the expression of markers associated with different stages of cardiogenic differentiation, including markers of early cardiogenic commitment, such as GATA binding protein 4 (GATA4), NK2 homeobox 5 (NKX2-5), and myocyte enhancer factor 2 C (MEF2C), as well as markers associated with cardiomyocyte maturation, including cardiac troponin I (cTnI), cardiac troponin T (cTnT/TNNT2), connexin 43 (Cx43), α-actinin-2 (ACTN2), desmin, sarcomeric actin, and α/β-myosin heavy chains (α-MHC and β-MHC). Studies evaluating the acquisition of a cardiac phenotype were also considered eligible. In the absence of a standardized marker panel for confirming cardiogenic differentiation of mesenchymal stem cells, studies evaluating at least one marker of early cardiogenic commitment, cardiomyocyte maturation, or acquisition of a cardiac phenotype were included.

Studies involving human participants, cell types other than mesenchymal stem cells, cardiogenic differentiation induced by strategies other than in vitro electrical stimulation, or studies that did not evaluate cardiogenic differentiation were considered ineligible. After duplicate removal, titles and abstracts were independently screened by two reviewers using Rayyan QCRI. Potentially eligible studies underwent independent full-text assessment according to the predefined eligibility criteria. When full-text articles were not available through open access, the corresponding authors were contacted by email to request access; articles that could not be retrieved were excluded. Disagreements were resolved through discussion and consensus.

This review was conducted and reported in accordance with the Preferred Reporting Items for Systematic Reviews and Meta-Analyses (PRISMA) 2020 statement. The PRISMA 2020 checklist is provided in Online Resource 2.

### Data extraction and analysis

A standardized data extraction form was developed to collect and summarize the relevant information from each included study. Extracted variables included MSC source, stimulation platform characteristics, electrical stimulation parameters, stimulation duration, methods used to assess cell viability and cardiogenic differentiation, and the main findings of each study.

The extracted data were synthesized narratively because substantial methodological heterogeneity was identified across the included studies in experimental design, electrical stimulation protocols, and outcome measures, precluding quantitative synthesis. Discordant findings were interpreted qualitatively according to study characteristics, including cell source, stimulation parameters, stimulation duration, and assessment methods. No weighting based on methodological quality or effect size was performed.

### Quality assessment

Because no validated tool is currently available to assess in vitro bioengineering studies involving electrical stimulation methodologically (Tran et al. 2021), an ad hoc quality assessment checklist was developed for this review. The checklist was developed in accordance with recommendations for reviews of in vitro studies (Tran et al. 2021) and the manuscript evaluation criteria proposed by the Academic Medicine and GEA-RIME committee (Bordage et al. 2001).

Accordingly, a formal risk-of-bias assessment was not performed. Instead, the checklist was intended to provide a structured assessment of methodological reporting and experimental rigor. It was therefore used to describe the completeness and rigor of the methods reported across the included studies and to improve transparency in study appraisal. The assessment criteria are described below, and their corresponding columns are presented in Table 1.

1. Problem statement, conceptual framework, and research question.
2. Bibliographic references and documentation.
3. Relevance.
4. Experimental design.
5. Instrumentation, data collection, and quality control.
6. Population and sample.
7. Description of the experimental system.
8. Statistical analysis.
9. Presentation of results.
10. Discussion and conclusions: interpretation.
11. Title, authors, and abstract.
12. Scientific behavior.

**Table 1:** Assessment of methodological reporting of the included studies using an ad hoc checklist

Publication	Categories of the review criteria checklist											
	1	2	3	4	5	6	7	8	9	10	11	12
Genovese et al. (2009a)	✓	✓	✓	✓	✓	✓		✓	✓	✓	✓	✓
Genovese et al. (2009b)	✓	✓	✓	✓	✓	✓		✓	✓	✓	✓	✓
Tandon et al. (2009)	✓	✓	✓	✓	✓	✓	✓	✓	✓	✓	✓	✓
Tandon et al. (2010)	✓	✓	✓	✓	✓	✓	✓	✓	✓	✓	✓	✓
Haneef et al. (2012)	✓	✓	✓	✓	✓	✓	✓	✓	✓	✓	✓	✓
Mooney et al. (2012)	✓	✓	✓	✓	✓	✓	✓	✓	✓	✓	✓	✓
Wen et al. (2013)	✓	✓	✓	✓	✓	✓	✓	✓	✓	✓	✓	✓
Wang et al. (2013)	✓	✓	✓	✓	✓	✓	✓	✓	✓	✓	✓	✓
Thrivikraman et al. (2016)	✓	✓	✓	✓	✓	✓	✓	✓	✓	✓	✓	✓
Tang et al. (2015)	✓	✓	✓	✓	✓	✓	✓	✓	✓	✓	✓	✓
He et al. (2019)	✓	✓	✓	✓	✓	✓	✓	✓	✓	✓	✓	✓
Herrmann et al. (2018)	✓	✓	✓	✓	✓	✓	✓	✓	✓	✓	✓	✓
Mombini et al. (2019)	✓	✓	✓	✓	✓	✓	✓	✓	✓	✓	✓	✓
Dat et al. (2020)	✓	✓	✓	✓	✓	✓	✓	✓	✓	✓	✓	✓
Moghadam et al. (2026)	✓	✓	✓	✓	✓	✓	✓	✓	✓	✓	✓	✓

Note: The checklist evaluates the completeness of methodological reporting and should not be interpreted as a formal assessment of study quality or risk of bias

## Results

**Fig. 1:**
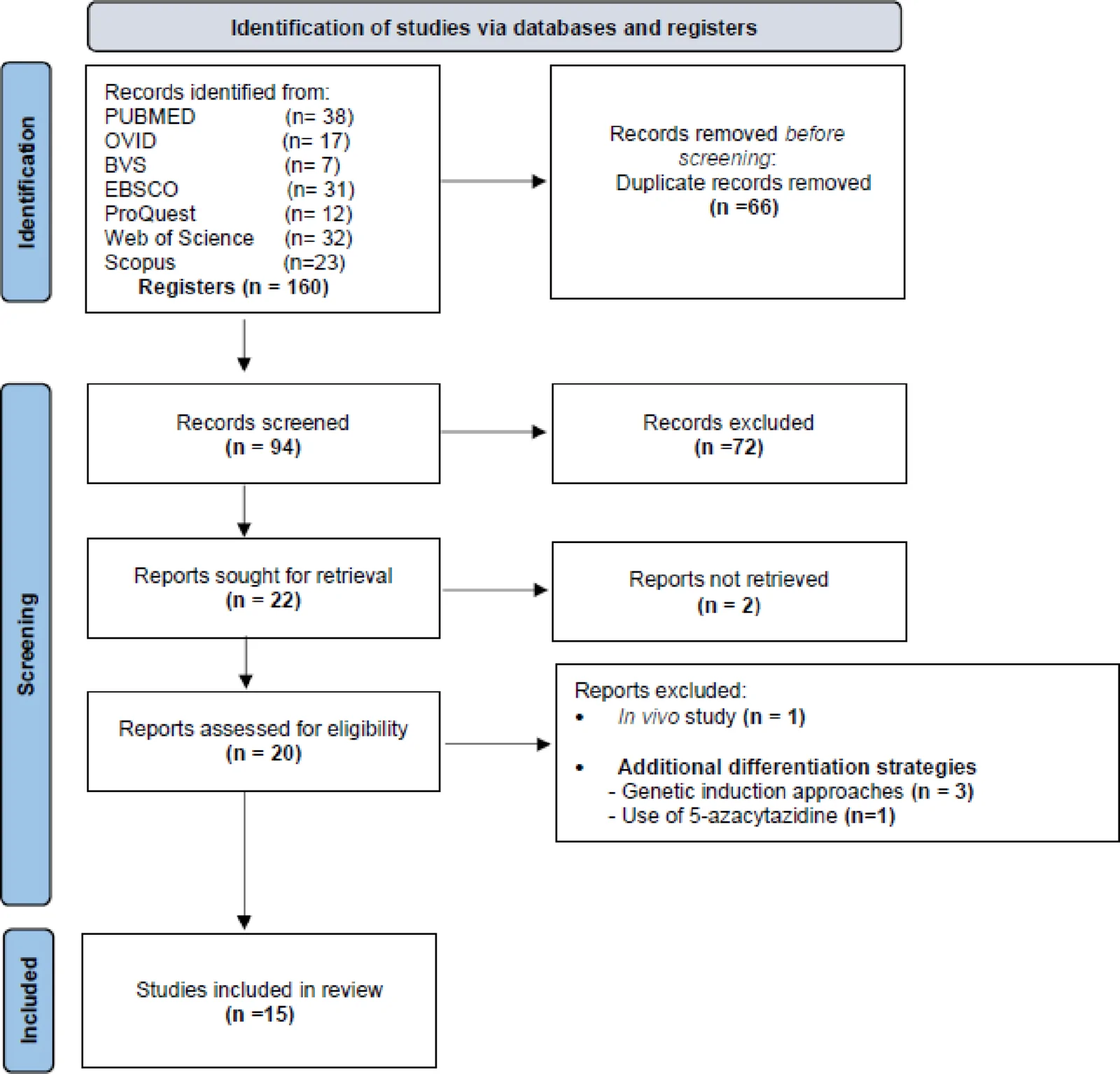
Article selection process according to the PRISMA 2020 statement. The flow diagram summarizes the identification, screening, eligibility assessment, and inclusion of studies evaluating the effects of electrical stimulation on the cardiogenic differentiation of mesenchymal stem cells

The updated literature search, last conducted on July 9, 2026, identified 160 records across the seven databases (Fig. 1). After removing 66 duplicate records, 94 studies remained for title and abstract screening. Seventy-two records were excluded according to the predefined eligibility criteria, and the detailed reasons for exclusion are summarized in the table provided in Online Resource 3. Of the remaining 22 reports sought for retrieval, two could not be obtained. Consequently, 20 articles underwent full-text assessment for eligibility, of which five were excluded because they involved in vivo electrical stimulation (*n* = 1) or employed additional differentiation strategies, including genetic induction approaches (*n* = 3) and the use of 5-azacytidine (*n* = 1). Ultimately, 15 studies met the eligibility criteria and were included in the review.

### Assessment of methodological reporting

The methodological reporting of the 15 included studies was assessed using the 12-item checklist described above. As shown in Table 1, most checklist items were reported across the included studies. Thirteen of the fifteen studies (86.7%) fulfilled all checklist criteria, whereas two studies (13.3%) fulfilled 11 of the 12 criteria. Criterion 7 (description of the experimental system) was the only checklist item not fulfilled by all studies and was reported in 13 studies (86.7%). The principal characteristics and findings of the included studies are presented in Table 2.

### Cellular models

**Fig. 2:**
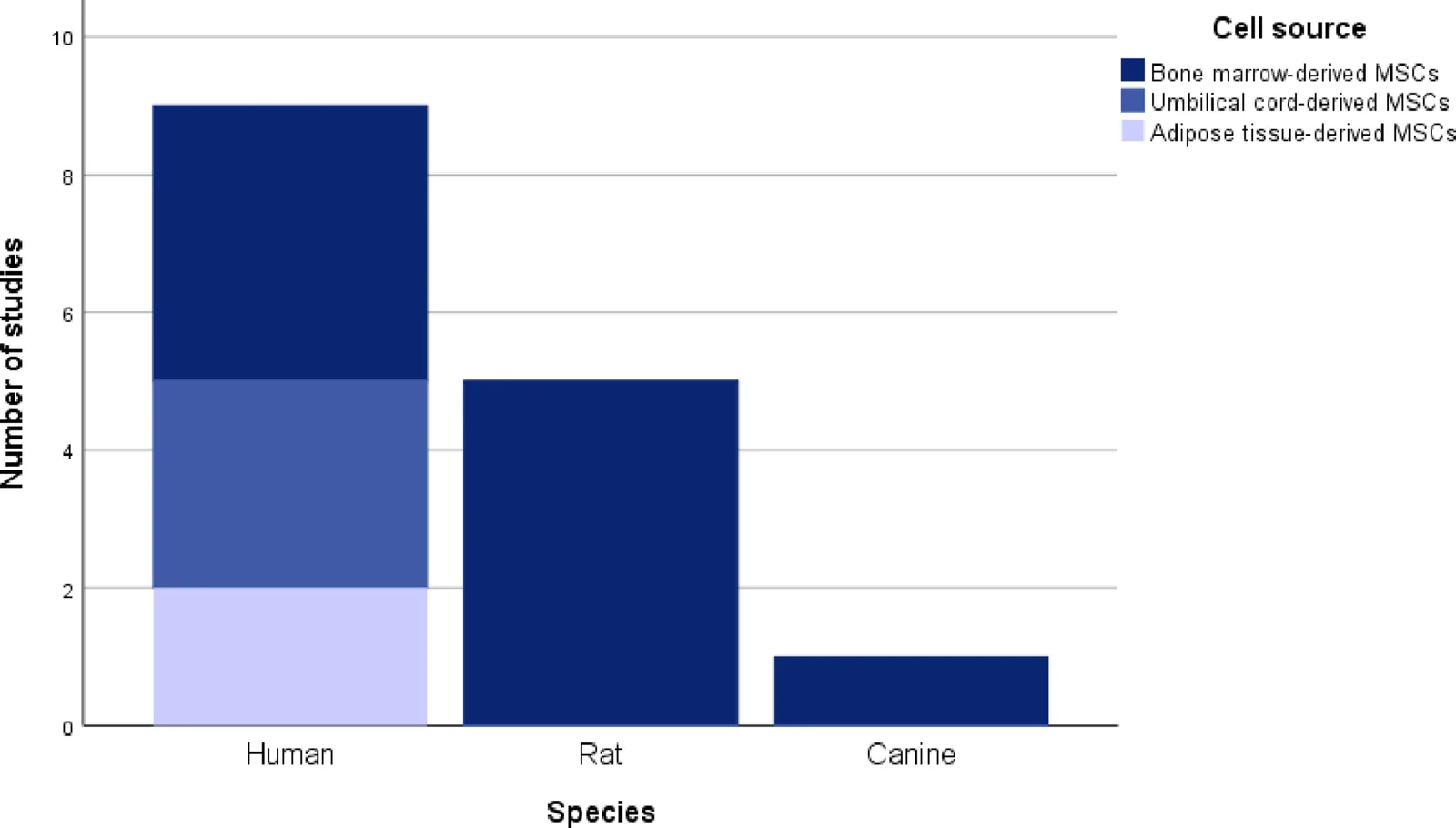
Distribution of mesenchymal stem cell species and tissue sources reported in the included studies. Studies were classified by species of origin (human, rat, and canine) and tissue source of mesenchymal stem cells (bone marrow, adipose tissue, and umbilical cord). Bars represent the number of included studies (*n* = 15), with each publication considered an independent unit of analysis. Cell source categories were assigned according to the information reported in the original articles

Among the 15 studies included in this review, 9 used human mesenchymal stem cells (MSCs), 5 employed rat-derived MSCs, and 1 used canine-derived MSCs. Human-derived MSCs were the most frequently investigated cell source overall (*n* = 9), including human bone marrow-derived MSCs (hBMSCs, *n* = 4), human umbilical cord-derived MSCs (hUC-MSCs, *n* = 3), and human adipose-derived stem cells (hASCs, *n* = 2). All rat studies used bone marrow-derived MSCs (rBMSCs, *n* = 5), whereas the single canine study employed canine bone marrow-derived MSCs (cBMSCs) (Fig. 2).

**Table 2:** Studies on MSC cardiogenic differentiation via electrical stimulation

Author	Cell type	Stimulation platform	Stimulation parameters	Total stimulation time	Assessment methods	Main findings
Genovese et al. 2009a (USA)	hBMSCs	C-Pace chronic stimulation unit (IonOptics)	10–40 V; 0.5 Hz; 5 ms	12 h	Trypan blue Live/Dead ICC Confocal IF WB Flow cytometry	↓ Cell viability; elongated fusiform morphology and alignment; myofibril reorganization; ↑ follistatin expression and short-term cardiogenic differentiation.
Genovese et al. 2009b (USA)	hBMSCs	C-Pace chronic stimulation unit (IonOptics)	10 V; 0.5 Hz; 5 ms	3 weeks	Trypan blue Live/Dead ICC Confocal IF WB	↓ Cell Viability; elongated and aligned morphology; multinucleation; ↑ cTnI, desmin, and Cx43 expression; improved fibrillar organization.
Tandon et al. 2009 (USA)	hASCs	Bioreactor with salt bridges and Ag–AgCl electrodes	3 mA; 0 Hz; DC	2–4 h	ICC IF RT‒qPCR	Cell alignment after 4 h; transient ↑ Cx43 expression followed by a decrease after 4 h.
Tandon et al. 2010 (USA)	hASCs	Bioreactor with indium and tin oxide electrodes	20 mv; 1 Hz; 2 ms Monophasic pulse	6 days	IF	↑ Gap-junction formation; gap junctions exhibited a preferential orientation perpendicular to the applied electric field.
Haneef et al. 2012 (France)	rat BMSCs	Bipolar pacemakers with curved needle electrodes	7 V; 2 Hz; 5 ms	3 weeks	MTT Confocal IF	↑ Cell proliferation ↑ cTnI, Cx43, sarcomeric actin, desmin, fast myosin, and slow myosin expression.
Mooney et al. 2012 (Ireland)	hBMSCs	Electrophysiological bioreactor with carbon nanotube scaffold	0,15 V/cm; 1 Hz; 2 ms	2 weeks	IF RT‒qPCR WB	Cell reorientation and fusiform morphology ↑ Nkx2.5, GATA4, cTnT, Cx43 and MHC expression.
Wen et al. 2013 (China)	canine BMSCs	Custom chamber with platinum electrodes	40 uA; 2 Hz; 2 ms Monophasic pulse	(1, 3 and 6 h/day) over 5 days	Confocal IF WB	↑ Cx43 and cTnI expression, greatest after 6 h/day stimulation; no significant changes in GATA4 or MEF2C expression
Wang et al. 2013 (USA)	rat BMSCs	Bioreactor with silver electrodes	5 V; 1 Hz; 10 ms	2.5 weeks	Live/Dead Confocal IF	↑ Cell density; no obvious alignment with electrical stimulation alone; combined stimulation improved morphology and alignment.
Thrivikraman et al. 2016(India)	hBMSCs	Custom chamber with stainless steel electrodes	Protocol 1: 100 mV/cm (continuous current) Protocol 2: 100 mV/cm; 1 Hz	(15 min/day) for 2 weeks	IF RT‒qPCR Flow cytometry	↑ GATA4 and cTnT expression; no significant changes in ACTN2.
Tang et al. 2015 (China)	rat BMSCs	Custom chamber with carbon electrodes	2 V; 2 Hz; 5 ms	(0.5, 2, 4 and 6 h/day) over 10 days	RT‒qPCR WB	↑ MEF2C and Cx43 expression The 2 h/day stimulation protocol produced the most favorable response.
He et al. 2019 (China)	rat BMSCs	Custom chamber with carbon electrodes	2 V; 2 Hz; 5 ms	(0.5, 2, 4 and 6 h/day) over 10 days	RT‒qPCR WB	Altered morphology with flattened cells, disrupted organization, and partial spindle-shaped morphology ↑ Cx43, TNNT2, ACTN2 and MEF2C expression.
Herrmann et al. 2018 (Germany)	hUC-MSCs	Bioreactor	3 V; 60 Hz	72 h	SYTO 9-PI Flow cytometry SEM	Dense cell layer formation with cellular extensions and intercellular connections.
Mombini et al. 2019 (Iran)	rat BMSCs	Polyvinyl acetate nanofibers modified with carbon nanotubes	0.005 V/cm; 1 Hz	20 days	MTT RT‒qPCR	Higher carbon nanotube concentrations were associated with ↓ cell viability and ↑ Nkx2.5, cTnI and β-MHC expression.
Dat et al. 2020 (Vietnam)	hUC-MSCs	Custom chamber with carbon electrodes	5 V/cm; 2 Hz; 5 ms Monophasic pulse	(10 min/day) during 15 days	Trypan blue ICC RT‒qPCR	Elongated and fusiform morphology; viability unaffected by stimulation ≤ 10 min/day; ↑ Nkx2.5, α-MHC, desmin, GATA4 and cTnI expression.
Moghadam et al. 2026 (Iran)	hUC-MSCs	Bioreactor with carbon electrodes	50 mV/cm; 1 Hz; 2 ms	2 weeks	Live/Dead MTT SEM RT-qPCR IF	Viability unaffected; elongated morphology and cytoskeletal organization; ↑ GATA4, cTnT, α-actin and Cx43 expression.

hBMSC: human mesenchymal stem cells from bone marrow; hASC: mesenchymal stem cells derived from adipose tissue; hUC-MSCs: human umbilical cord-derived mesenchymal stem cells; ICC: immunocytochemistry; IF: immunofluorescence; WB: Western blot; RT-qPCR: reverse transcription-quantitative polymerase chain reaction; MTT: 3-(4,5-dimethylthiazol-2-yl)-2,5-diphenyltetrazolium bromide assay; SYTO 9-PI: propidium iodide staining with SYTO9; SEM: scanning electron microscopy cTnI: cardiac troponin I; cTnT: cardiac troponin T Cx43: connexin 43; Nkx2.5: transcription factor Nkx2.5; GATA4: transcription factor GATA4; CMHC: cardiac myosin heavy chain; S100A4: S100-A4 protein; MEF2C: transcription factor MEF2C; ACTN2: actinin alpha 2; TNNT2: cardiac troponin T type 2; TGFβ1: transforming growth factor beta 1; α-MHC: myosin heavy alpha chain

### Experimental platforms

The experimental platforms used to deliver electrical stimulation for the in vitro cardiogenic differentiation of mesenchymal stem cells were identified across the included studies. Custom culture chambers were the most frequently employed platform, accounting for seven of the fifteen studies (46.6%) (Table 3). Bioreactors were used in six studies (40.0%), and a direct stimulation strategy based on surface electrodes was reported in one study (6.7%). Similarly, one study (6.7%) employed an electroconductive scaffold fabricated by electrospinning, which, in addition to providing structural support, served as an electrical conductor.

### Experimental materials

The conductive electrode material was reported in 14 of the 15 included studies. Carbon-based electrodes were the most commonly reported material (6/15, 40.0%), followed by silver-based electrodes, including silver (Ag) and silver/silver chloride (Ag/AgCl), reported in four studies (26.7%) (Table 3). Platinum and stainless-steel electrodes were each used in two studies (13.3%), whereas indium tin oxide (ITO) was reported in one study (6.7%). One publication did not report the conductive electrode material.

**Table 3:** Distribution of electrical stimulation platforms and conductive electrode materials across the included studies

Electrical stimulation platform	Carbon	Silver*	Platinum	Stainless steel	ITO	NR	Total
Custom culture chamber	3	2	1	1	0	0	7
Bioreactor	2	2	0	0	1	1	6
Surface electrodes	0	0	1	0	0	0	1
Electroconductive scaffold	1	0	0	0	0	0	1
Total	6	4	2	1	1	1	**15**

Numbers indicate the number of studies reporting each combination of electrical stimulation platform and conductive electrode material. *Silver includes both silver (Ag) and silver/silver chloride (Ag/AgCl) electrodes. ITO: Indium tin oxide, NR: not reported

### Electrical stimulation protocols

The stimulation amplitude was reported using different units depending on the study design (Table 2). Absolute voltage (mV or V) was the most frequently used approach (53.3%), followed by normalized voltage (V/cm) (33.3%) and current intensity (µA or mA) (13.3%). Absolute voltage ranged from 20 mV to 10 V, normalized voltage from 0.005 to 5 V/cm, and current intensity from 40 µA to 3 mA. One study evaluated two stimulation protocols using normalized voltage. Owing to these differences in reporting units, direct comparison of stimulation amplitudes across studies was not possible.

Pulsed electrical stimulation was the predominant waveform. The reported stimulation frequencies ranged from 0.5 to 60 Hz, with 1 Hz being the most frequently used frequency (40.0%), followed by 2 Hz (33.3%). Lower frequencies (0.5 Hz) and high-frequency stimulation (60 Hz) were each reported in a single study. Among the 11 studies reporting pulse duration, 5-ms pulses were the most frequently used (54.5%), followed by 2-ms pulses (36.4%) and 10-ms pulses (9.1%).

The total duration of electrical stimulation varied substantially, ranging from 2 h to 3 weeks. Nevertheless, 2-week stimulation protocols were the most frequently reported (26.7%). Some studies applied continuous stimulation, whereas others employed fractionated protocols consisting of repeated daily stimulation periods ranging from 10 min/day to 6 h/day over several days or weeks. This considerable methodological heterogeneity highlights the lack of standardized electrical stimulation protocols for cardiogenic differentiation of MSCs.

### Experimental techniques

The included studies primarily assessed cardiogenic differentiation using molecular and phenotypic approaches, including immunocytochemistry, immunofluorescence, reverse transcription quantitative polymerase chain reaction (RT–qPCR), Western blotting, and flow cytometry. However, comprehensive functional assessments, such as electrophysiological recordings, calcium transient analyses, or contractility measurements, were largely absent.

**Fig. 3:**
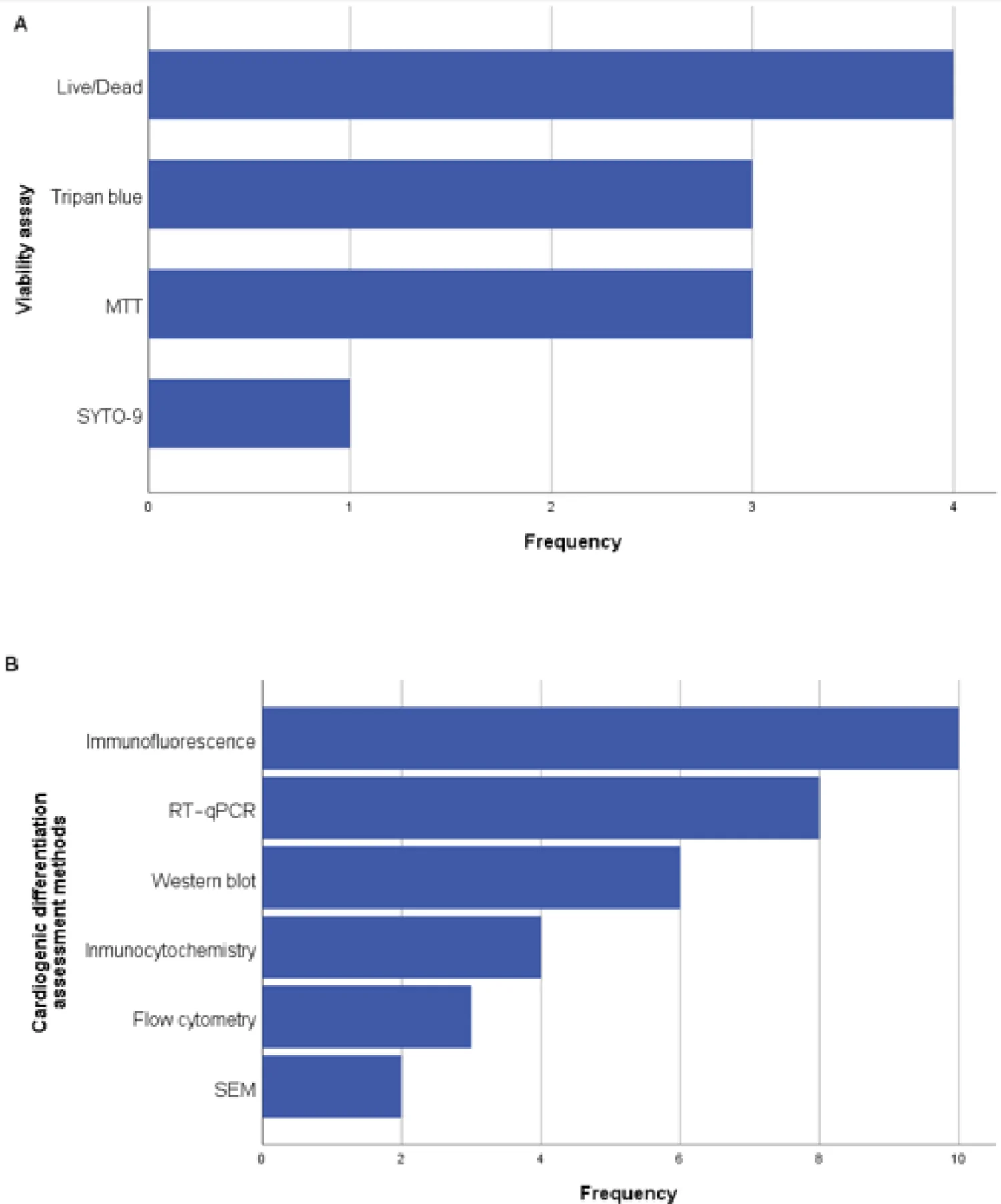
Experimental techniques used to assess cell viability and cardiogenic differentiation across the included studies. (a) Experimental techniques used to evaluate cell viability. (b) Experimental techniques used to evaluate cardiogenic differentiation. Frequencies indicate the number of included studies that reported each analytical technique. Studies using more than one assessment method contributed to multiple categories; therefore, the total frequency exceeds the number of included studies. Techniques were classified according to the methodology described in each original publication

Cell viability was evaluated in 8 of the 15 included studies using four different analytical methods (Fig. 3a). Live/Dead assays were the most frequently reported, being used in four studies (50.0%), followed by Trypan blue and MTT assays, each reported in three studies (37.5%). The SYTO9/PI assay was used in one study (12.5%). Several studies employed more than one viability assay; therefore, the total frequency of reported methods exceeds the number of studies that assessed cell viability.

Cardiogenic differentiation was primarily evaluated by immunofluorescence, which was reported in 10 of the 15 studies (66.7%), followed by RT–qPCR (8 studies, 53.3%), Western blot (6 studies, 40.0%), immunocytochemistry (4 studies, 26.7%), and flow cytometry (3 studies, 20.0%) (Fig. 3b). These techniques were mainly used to assess the expression of cardiac-specific proteins and genes associated with cardiogenic commitment and maturation.

### Experimental results

**Fig. 4:**
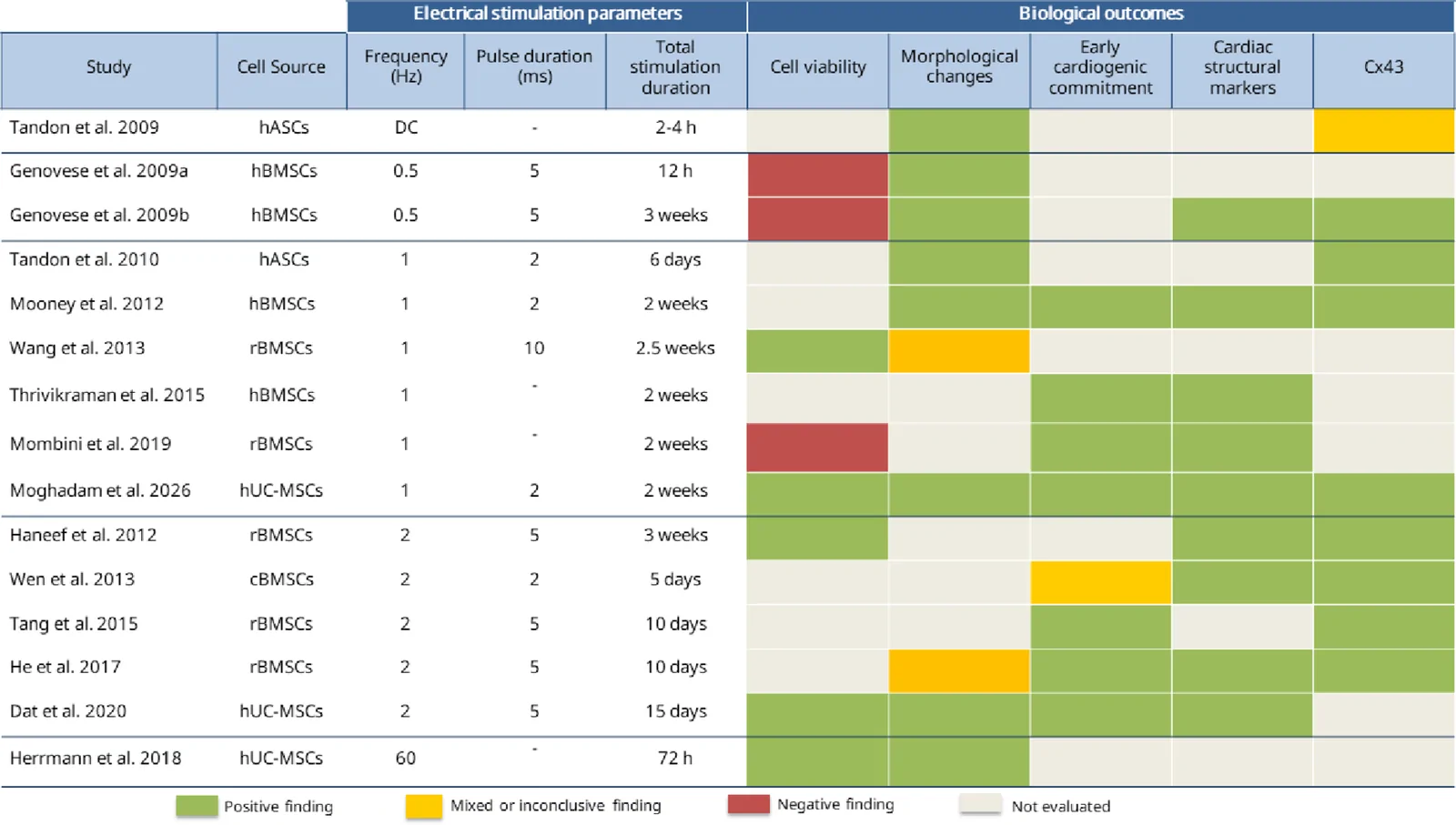
Heatmap-style evidence matrix summarizing electrical stimulation parameters and reported biological findings across the included studies. Studies are grouped according to stimulation frequency to facilitate visual comparison of the experimental protocols and their reported biological findings. Colors indicate the reported findings for each biological outcome: green, positive finding (e.g., increased marker expression, preserved cell viability, or favorable morphological changes); yellow, mixed or inconclusive findings (e.g., inconsistent responses among evaluated outcomes or no clear overall change); red, negative finding (e.g., reduced cell viability or unfavorable biological response); gray, outcome not evaluated. Early cardiogenic commitment refers to the evaluation of at least one early cardiogenic marker (GATA4, NKX2.5, or MEF2C). Cardiac structural markers include at least one of the following: cTnI, cTnT (TNNT2), ACTN2, desmin, α-MHC, β-MHC, sarcomeric actin, fast myosin, or slow myosin. Cx43 indicates connexin 43 expression. ASCs, adipose-derived mesenchymal stem cells; hBMSCs, human bone marrow-derived mesenchymal stem cells; rBMSCs, rat bone marrow-derived mesenchymal stem cells; cBMSCs, canine bone marrow-derived mesenchymal stem cells; hUC-MSCs, human umbilical cord-derived mesenchymal stem cells; DC, direct current

Eight of the 15 included studies evaluated cell viability using the previously described assays (Fig. 4). Overall, five studies reported preserved cell viability following electrical stimulation. In contrast, three studies reported reduced cell viability under the experimental conditions used. The negative findings were limited to specific experimental settings, while most stimulation protocols maintained cell viability throughout the differentiation process.

Morphological changes associated with cardiogenic differentiation were evaluated in ten studies and were predominantly characterized by cell elongation, spindle-shaped morphology, and cellular alignment in response to electrical stimulation. Overall, eight studies reported positive morphological findings, whereas two studies presented mixed or inconclusive findings. These included increased cell density without evident alignment in one study, and altered morphology characterized by flattened cells, disruption of the organized cellular pattern, and the presence of some spindle-shaped cells in another study (Fig. 4).

Electrical stimulation generally promoted early cardiogenic commitment, as evidenced by increased expression of at least one early cardiogenic marker (GATA4, NKX2.5, or MEF2C) in most studies evaluating these markers. Only one study reported mixed or inconclusive findings: no significant changes in GATA4 or MEF2C expression were observed in MSCs, and no study reported an overall negative effect on early cardiogenic commitment.

Similarly, cardiac structural markers were predominantly reported as positive findings following electrical stimulation (Fig. 4). Increased expression of proteins associated with cardiomyocyte structural maturation—including cTnI, cTnT (TNNT2), desmin, α-MHC, β-MHC, sarcomeric actin, fast myosin, and slow myosin—was reported in the studies in which these markers were evaluated. Overall, most studies reported increased expression of markers associated with cardiac commitment, structural maturation, and intercellular coupling following electrical stimulation.

Among the evaluated biomarkers, Cx43 was the most frequently assessed (60.0%, 9/15), followed by cTnI (33.3%, 5/15), whereas cTnT (TNNT2) and GATA4 were each evaluated in 26.7% (4/15) of the studies and NKX2.5 in 20.0% (3/15). In addition to increased Cx43 expression, one study reported enhanced gap-junction formation and preferential orientation of gap junctions following electrical stimulation, suggesting improved electrical coupling between adjacent cells. Another study, however, reported mixed findings, with an initial increase in Cx43 expression followed by a reduction after prolonged electrical stimulation.

In contrast, the findings for ACTN2 and MEF2C were less consistent. Whereas one study reported increased ACTN2 expression, another observed no significant changes. Similarly, two studies reported increased MEF2C expression, whereas another found no significant changes in either MEF2C or GATA4 following electrical stimulation.

## Discussion

The search strategy and review question were designed to identify a focused and thematically specific body of evidence. Despite an exhaustive search across seven major bibliographic databases, relatively few eligible studies were identified, indicating that this remains an emerging area of research. Nevertheless, the ongoing publication of studies during the search period suggests that research on the application of electrical stimulation to cardiogenic differentiation of MSCs remains active.

### Origin and diversity of MSCs

The predominance of human cellular models likely reflects the aim of generating findings more relevant to human biology. In contrast, the frequent use of bone marrow-derived MSCs is consistent with the historical role of bone marrow as the primary source of mesenchymal stem cells (Pittenger et al. 1999). However, bone marrow harvesting is an invasive procedure, underscoring the need to investigate more accessible MSC sources, such as adipose tissue, which remained underrepresented in the included studies despite its higher cell yield and easier collection (Fraser et al. 2006). In addition to human cells, several studies employed animal-derived MSCs, predominantly from rat bone marrow and, less frequently, from canine sources. Although these models are widely used in preclinical research, canine MSCs may also offer opportunities to explore applications relevant to veterinary regenerative medicine. Nevertheless, species-specific differences in MSC biology—including proliferative capacity, differentiation potential, electrophysiological properties, and responsiveness to biophysical stimuli—may have contributed to the variability observed across studies (Scuteri et al. 2014; Vieira et al. 2010). Consequently, the effects of electrical stimulation should not be directly extrapolated between species, as these biological differences represent an additional source of heterogeneity that limits direct comparisons and the identification of standardized stimulation protocols.

### Variation in electrical stimulation parameters

Electrical stimulation protocols varied substantially across the included studies with respect to waveform, amplitude, frequency, pulse duration and total stimulation time. This methodological heterogeneity limited direct comparisons among studies and remains one of the main obstacles to establishing standardized stimulation protocols for cardiogenic differentiation of MSCs.

Frequency was one of the most consistent stimulation parameters across the included studies, with most protocols employing low frequencies (1–2 Hz), comparable to the resting heart rate of human cardiomyocytes (≈ 1–1.67 Hz). These frequencies may enhance intracellular calcium signaling, thereby promoting cardiomyocyte contraction, sarcomeric maturation, and gap junction formation (Au et al. 2007; Radisic et al. 2008). In contrast, higher frequencies have produced less consistent effects (Fujita et al. 2007). However, because several studies used rat-derived MSCs, whose native cardiac frequency is substantially higher (≈ 5 Hz), stimulation frequency should be selected according to the physiological characteristics of the species under investigation rather than assuming a universal protocol (Chan et al. 2010).

Pulsed current predominated among the included studies, with pulse widths of approximately 5 ms being the most frequently reported. Pulse duration may prolong membrane depolarization and facilitate activation of voltage-dependent Ca²⁺ channels, thereby promoting calcium-dependent signaling pathways involved in cardiac gene expression (Nguemo et al. 2013). In addition, pulsed stimulation facilitates charge dissipation between pulses, reducing electrode polarization (Thrivikraman et al. 2018). However, pulse duration should not be considered in isolation, as its biological effects depend on interactions with stimulation frequency, amplitude, and total stimulation time.

Although pulsed stimulation was consistently employed, detailed pulse characteristics were reported inconsistently across the included studies. Only three studies explicitly reported the use of monophasic pulses, precluding comparisons between pulse configurations and their potential influence on cardiogenic differentiation outcomes. The broader bioelectrical stimulation literature generally favors biphasic pulses because monophasic stimulation generates a net charge that accumulates at the electrode surface, promoting faradaic reactions and the formation of electrochemical by-products that may compromise cell viability, whereas biphasic pulses minimize charge accumulation and improve electrochemical stability (Chiu et al. 2011; Piallat et al. 2009; Pietronave et al. 2014). Nevertheless, the evidence identified in this review remains insufficient to determine whether a specific pulse configuration provides superior biological outcomes for promoting cardiogenic differentiation of MSCs.

Stimulation amplitude was one of the most heterogeneous parameters across studies. The wide variation in amplitude values, together with inconsistent reporting units and incomplete descriptions of key experimental variables such as interelectrode distance, limited direct comparisons and prevented the identification of an optimal electrical dose. These findings underscore the importance of standardized reporting of stimulation parameters in future studies.

Under physiological conditions, cardiomyocytes depolarize following relatively small changes in membrane potential (Scott et al. 2022). Consistent with this concept, Tandon et al. (2010) demonstrated that a 20 mV potential difference enhanced cell proliferation and gap junction expression. However, most studies included in this review applied substantially higher stimulation amplitudes than those observed in native cardiac tissue. This difference likely reflects the characteristics of in vitro culture systems, in which the effective electrical stimulus is influenced by medium conductivity, electrode configuration, and the spatial distribution of the electric field, often requiring higher applied amplitudes to elicit measurable cellular responses. However, reports of reduced cell viability under prolonged stimulation or relatively high amplitudes should be interpreted with caution, as they stem from heterogeneous experimental models employing different stimulation units and protocols. Consequently, no universal amplitude threshold for cardiogenic differentiation can currently be established.

The duration of electrical stimulation also varied considerably among studies and was associated with differences in the reported cardiogenic outcomes. Short stimulation periods (hours) were primarily associated with early morphological changes. In contrast, studies employing longer protocols (weeks) more frequently reported expression of cardiac proteins associated with structural maturation and intercellular coupling, including troponin and connexin 43. However, because stimulation duration was closely linked to differences in cell source, stimulation parameters, and experimental design, these observations should be interpreted with caution.

Overall, this systematic review identified the electrical stimulation parameters most frequently used to promote cardiogenic differentiation of MSCs. Frequencies of approximately 1 Hz, pulse widths of 5 ms, and stimulation periods of about two weeks were the conditions most commonly employed. However, because these parameters were evaluated using heterogeneous experimental designs and were rarely compared directly, they should not be interpreted as evidence of an optimal stimulation protocol. Instead, they provide a useful reference for the design of future studies, which should include systematic optimization of stimulation parameters, particularly amplitude, through preliminary dose–response experiments.

### Influence of the experimental platform on stimulation outcomes

Carbon-based electrodes were the most frequently used conductive material among the included studies. However, this finding should not be interpreted as evidence that carbon is superior to other conductive materials, as no studies have directly compared electrode materials under equivalent experimental conditions. In addition to carbon, silver was used as an electrode material in a limited number of studies because of its high electrical conductivity and low electrical resistance. However, under continuous electrical stimulation, silver may undergo surface oxidation, releasing Ag⁺ ions that can alter the culture microenvironment and affect cell viability (Bard et al. 2001). Although Ag/AgCl electrodes provide stable electrochemical behavior as reference electrodes in electrophysiological applications, their use as active stimulation electrodes requires careful consideration because of the potential for ionic interference and electrochemical instability (Merrill et al. 2005).

Beyond the selection of stimulation parameters, the characteristics of the experimental platform may also contribute substantially to the variability observed among studies. Differences in electrode geometry, interelectrode distance, conductive material, culture medium impedance, and the incorporation of three-dimensional matrices modify the spatial distribution of the electric field and consequently the effective electrical stimulus experienced by the cells (Merrill et al. 2005). These platform-specific characteristics limit reproducibility and complicate direct comparisons among studies.

### Biological responses to electrical stimulation

Among the eight studies that evaluated cell viability, five reported preserved viability following electrical stimulation, whereas three described reduced viability under specific experimental conditions. These findings suggest that the biological effects of electrical stimulation depend on both stimulation parameters and the characteristics of the experimental platform, rather than reflecting a uniform response. Whether the observed reduction in viability reflects stimulation-induced cytotoxicity or the selective survival of cells undergoing cardiogenic commitment remains unclear. In the former case, further optimization of stimulation parameters would be required, as longer pulse durations have been associated with increased apoptosis at equivalent electric field intensities (Prado et al. 2016). Similarly, Genovese et al. suggested that stimulation amplitudes of ≥ 10 V may adversely affect MSC viability, highlighting the importance of optimizing stimulation parameters before their implementation in cardiogenic differentiation studies.

Differences in outcome assessment further limited comparisons of cell viability across studies. While some studies directly evaluated viability using assays such as MTT, Live/Dead, Trypan Blue, or SYTO9/PI staining, others inferred viability from increases in cell number or proliferation without performing specific viability assays. Because cell viability and cell proliferation represent related but distinct biological processes, these outcomes should not be considered equivalent. This methodological heterogeneity further limits comparisons among studies and emphasizes the need for standardized approaches to evaluate the effects of electrical stimulation on cell survival and proliferation.

Connexin 43 was the most frequently evaluated gap junction protein among the included studies. Several studies reported increased Cx43 expression and redistribution from a diffuse intracellular pattern toward regions of cell–cell contact, findings consistent with changes in intercellular coupling and structural progression toward a cardiac-like phenotype. However, in the absence of electrophysiological assessments, these findings should not be interpreted as evidence of functional electrical integration. Contractile proteins such as troponin T and troponin I were also evaluated as indicators of structural maturation, whereas transcription factors including Nkx2.5 and GATA4 were primarily associated with early cardiogenic commitment rather than cardiomyocyte maturation (Dal-Pra et al. 2019).

In contrast to the generally positive findings observed for other cardiac markers, ACTN2 and MEF2C showed inconsistent results across studies. These discrepancies were observed among studies that differed substantially in MSC source, stimulation parameters, stimulation duration, experimental platforms and analytical methods. These inconsistencies may partly reflect methodological heterogeneity among studies rather than uniform biological responses to electrical stimulation. Consequently, the available evidence remains insufficient to draw definitive conclusions about the effects of electrical stimulation on ACTN2 and MEF2C expression.

### Limitations

This review has several limitations. First, the available evidence was characterized by substantial heterogeneity in MSC source, electrical stimulation protocols, experimental platforms, and outcome measures, precluding quantitative synthesis and limiting direct comparisons among studies. Consequently, the magnitude and consistency of the reported effects could not be quantitatively assessed and the conclusions are based on a qualitative narrative synthesis that should be interpreted in the context of this methodological heterogeneity.

Second, cardiogenic differentiation was evaluated predominantly through morphological changes and cardiac marker expression, whereas comprehensive functional assessments of cardiomyocyte maturation were limited. Therefore, the reported findings should be interpreted as evidence of varying degrees of cardiogenic commitment and structural maturation rather than definitive proof of a mature and functional cardiomyocyte phenotype. Finally, a formal risk-of-bias assessment was not performed because no validated instrument is currently available for this type of in vitro study. Instead, an ad hoc checklist based on previously published recommendations was used to assess methodological reporting. Therefore, the checklist findings should be interpreted descriptively and not as evidence of low risk of bias or high methodological quality.

## Conclusions and Perspectives

This systematic review identified the electrical stimulation parameters most frequently investigated in studies of cardiogenic differentiation of mesenchymal stem cells and synthesized the available evidence in this field. Although frequencies of approximately 1 Hz, pulse durations of 5 ms, and stimulation periods of about two weeks were the most commonly employed conditions, the marked methodological heterogeneity among studies precludes the identification of an optimal stimulation protocol.

Overall, the available studies report changes in cardiac lineage marker expression and morphology following electrical stimulation, suggesting potential effects on cardiogenic commitment and structural maturation. However, the substantial methodological heterogeneity, inconsistent findings across some outcomes and limited functional characterization preclude definitive conclusions regarding the magnitude, consistency or functional significance of these effects.

Future studies should prioritize standardized experimental designs, direct comparisons of MSC sources and stimulation protocols, and comprehensive functional assessments. Mechanistic studies, including electrophysiological characterization and complementary omics approaches, may further elucidate the molecular pathways underlying electrically induced cardiogenic differentiation. From a translational perspective, additional preclinical studies are required to establish the safety, reproducibility, and efficacy of electrical stimulation protocols before their potential application in cardiac regenerative medicine.

## Supplementary Information

Below is the link to the electronic supplementary material.

Supplementary Material 1

Supplementary Material 2

Supplementary Material 3

## Data Availability

This study is a systematic review based on previously published studies. No primary data were generated; however, data were extracted, synthesized, and analyzed from existing research.

## References

[CR1] Abdi R, Fiorina P, Adra CN, Atkinson M, Sayegh MH (2008) Immunomodulation by mesenchymal stem cells: A potential therapeutic strategy for type 1 diabetes. Diabetes 57(7):1759–1767. 10.2337/db08-0180 PMC2453631 18586907

[CR2] Almeida SO, Skelton RJ, Adigopula S, Ardehali R (2015) Arrhythmia in stem cell transplantation. Cardiac Electrophysiol Clin 7(2):357–370. 10.1016/j.ccep.2015.03.012 PMC4441953 26002399

[CR3] Au HTH, Cheng I, Chowdhury MF, Radisic M (2007) Interactive effects of surface topography and pulsatile electrical field stimulation on orientation and elongation of fibroblasts and cardiomyocytes. Biomaterials 28:4277–4293. 10.1016/j.biomaterials.2007.06.001 PMC2039774 17604100

[CR4] Bard AJ, Faulkner LR (2001) Electrochemical methods: Fundamentals and applications, 2nd edn. Wiley. https://books.wiley.com/titles/9781119334064/

[CR5] Ben-David U, Benvenisty N (2011) The tumorigenicity of human embryonic and induced pluripotent stem cells. Nat Rev Cancer 11:268–277. 10.1038/nrc3034 21390058

[CR6] Bordage G, Caelleigh AS, Steinecke A, Bland CJ, Crandall SJ, McGaghie WC, Pangaro LN, Penn G, Regehr G, Shea JS (2001) Joint Task Force of Academic Medicine and the GEA-RIME Committee. Review criteria for research manuscripts. Acad Med 76(9):897–978 PMID: 11565558 11565558

[CR7] Chan Y-C, Tse H-F, Siu C-W, Wang K, Li RA (2010) Automaticity and conduction properties of bio-artificial pacemakers assessed in an in vitro monolayer model of neonatal rat ventricular myocytes. Europace 12(8):1178–1187. 10.1093/europace/euq120 PMC2910602 20472688

[CR8] Chiu LLY, Iyer RK, King JP, Radisic M (2011) Biphasic electrical field stimulation aids in tissue engineering of multicell-type cardiac organoids. Tissue Eng Part A 17(11–12):1465–1477. 10.1089/ten.tea.2007.0244 PMC3098954 18783322

[CR9] Dai Y, Mu J, Zhou F (2021) The use of electrical stimulation to induce cardiac differentiation of stem cells for the treatment of myocardial infarction. Rev Cardiovasc Med 22(4):1167–1171. 10.31083/j.rcm2204125 34957760

[CR10] Dal-Pra S, Hodgkinson CP, Dzau VJ (2019) Induced cardiomyocyte maturation: Cardiac transcription factors are necessary but not sufficient. PLoS ONE 14(10):e0223842. 10.1371/journal.pone.0223842 PMC6797484 31622977

[CR11] Dat NH, Minh LH, Len HT, Vinh LG, Ha CT (2020) Hue biostimulator induce differentiation of umbilical cord mesenchymal stem cells into cardiomyocyte-like cells. Vietnam Med J 1:11–23. https://www.researchgate.net/publication/350107109_Hue_Biostimulator_induce_differentiation_of_umbilical_cord_mesenchymal_stem_cells_into_cardiomyocyte-like_cells

[CR12] Eng G, Lee BW, Protas L, Gagliardi M, Brown K, Kass RS et al (2016) Autonomous beating rate adaptation in human stem cell-derived cardiomyocytes. Nat Commun 7:10312. 10.1038/ncomms10312 PMC4735644 26785135

[CR13] Fraser JK, Wulur I, Alfonso Z, Hedrick MH (2006) Fat tissue: An underappreciated source of stem cells for biotechnology. Trends Biotechnol 24:150–154. 10.1016/j.tibtech.2006.01.010 16488036

[CR14] Fujita H, Nedachi T, Kanzaki M (2007) Accelerated de novo sarcomere assembly by electric pulse stimulation in C2C12 myotubes. Exp Cell Res 313:1853–1865. 10.1016/j.yexcr.2007.03.002 17425954

[CR15] Genovese JA, Spadaccio C, Garcia Rivello H, Toyoda Y, Patel AN (2009a) Electrostimulated bone marrow human mesenchymal stem cells produce follistatin. Cytotherapy 11(4):448–456. 10.1080/14653240902960445 19530028

[CR16] Genovese JA, Spadaccio C, Chachques E, Schussler O, Carpentier A, Chachques JC, Patel AN (2009b) Cardiac pre-differentiation of human mesenchymal stem cells by electrostimulation. Front Biosci 14:2996–3002. 10.2741/3429 19273251

[CR17] Haneef K, Lila N, Benadda S, Legrand F, Carpentier A, Chachques JC (2012) Development of bioartificial myocardium by electrostimulation of 3D collagen scaffolds seeded with stem cells. Heart Int 7(1):e14. 10.4081/hi.2012.e14 PMC3504306 23185681

[CR18] Hare JM, Fishman JE, Gerstenblith G et al (2012) Comparison of allogeneic vs autologous bone marrow-derived mesenchymal stem cells delivered by transendocardial injection in patients with ischemic cardiomyopathy: The POSEIDON randomized trial. JAMA 308(22):2369–2379. 10.1001/jama.2012.25321 PMC4762261 23117550

[CR19] He X, Li L, Tang M, Zeng Y, Li H, Yu X (2019) Biomimetic electrical stimulation induces rat bone marrow mesenchymal stem cells to differentiate into cardiomyocyte-like cells via TGF-beta 1 in vitro. Prog Biophys Mol Biol 148:47–53. 10.1016/j.pbiomolbio.2017.09.023 28969971

[CR20] Herreros J, Chachques JC, Trainini J, Pontón A, Sarralde A, Genovese J (2011) Regeneración celular cardíaca. Cirugía Cardiovasc 18(3):207–215. 10.1016/S1134-0096(11)70056-0

[CR21] Herrmann FEM, Lehner A, Koenig F, Hollweck T, Fano C, Dauner M, Eissner G, Hagl C, Akra B (2018) A feasibility study of a multimodal stimulation bioreactor for the conditioning of stem cell seeded cardiac patches. Biomed Mater Eng 1:1–12. 10.3233/BME-181031 30530957

[CR23] Merrill DR, Bikson M, Jefferys JG (2005) Electrical stimulation of excitable tissue: Design of efficacious and safe protocols. J Neurosci Methods 141(2):171–198. 10.1016/j.jneumeth.2004.10.020 15661300

[CR24] Moghadam M, Khani M-M, Sotoodehnejadnematalahi F, Tafazzoli-Shadpour M (2026) A novel conductive poly (sorbitol sebacate)/polyaniline film to promote cardiomyogenic differentiation of mesenchymal stem cells under electrical stimulation. Reactive Funct Polym 219:106582. 10.1016/j.reactfunctpolym.2025.106582

[CR25] Mombini S, Mohammadnejad J, Bakhshandeh B, Narmani A, Nourmohammadi J, Vahdat S, Zira K (2019) Chitosan–PVA–CNT nanofibers as electrically conductive scaffolds for cardiovascular tissue engineering. Int J Biol Macromol 140:278–287. 10.1016/j.ijbiomac.2019.08.046 31400428

[CR26] Mooney E, Mackle JN, Blond DJ-P, O’Cearbhaill E, Shaw G, Blau WJ, Barry FP, Barron V, Murphy JM (2012) The electrical stimulation of carbon nanotubes to provide a cardiomimetic cue to MSCs. Biomaterials 33(27):6132–6139. 10.1016/j.biomaterials.2012.05.032 22681974

[CR27] Nguemo F, Fleischmann BK, Gupta MK, Sarić T, Malan D, Liang H, Pfannkuche K, Bloch W, Schunkert H, Hescheler J, Reppel M (2013) The L-type Ca2 + channels blocker nifedipine represses mesodermal fate determination in murine embryonic stem cells. PLoS ONE 8(1):e53407. 10.1371/journal.pone.0053407 PMC3539992 23320083

[CR28] Piallat B, Chabardès S, Devergnas A, Torres N, Allain M, Barrat E, Benabid AL (2009) Monophasic but not biphasic pulses induce brain tissue damage during monopolar high-frequency deep brain stimulation. Neurosurgery 64(1):156–162. 10.1227/01.NEU.0000336331.88559.CF 19145164

[CR29] Pietronave S, Zamperone A, Oltolina F, Colangelo D, Follenzi A, Novelli E, Diena M, Pavesi A, Consolo F, Fiore GB, Soncini M, Prat M (2014) Monophasic and biphasic electrical stimulation induces a precardiac differentiation in progenitor cells isolated from human heart. Stem Cells Dev 23(8):888–898. 10.1089/scd.2013.0375 PMC3991992 24328510

[CR31] Pittenger MF, Martin BJ (2004) Mesenchymal stem cells and their potential as cardiac therapeutics. Circul Res 95(1):9–20. 10.1161/01.RES.0000135902.99383.6f 15242981

[CR30] Pittenger MF, Mackay AM, Beck SC, Jaiswal RK, Douglas R, Mosca JD, Moorman MA, Simonetti DW, Craig S, Marshak D (1999) Multilineage potential of adult human mesenchymal stem cells. Science 284(5411):143–147. 10.1126/science.284.5411.143 10102814

[CR32] Prado LN, Goulart JT, Zoccoler M, Oliveira PX (2016) Ventricular myocyte injury by high-intensity electric field: Effect of pulse duration. Gen Physiol Biophys 35(2):121–130. 10.4149/gpb_2015047 26830130

[CR33] Radisic M, Fast VG, Sharifov OF, Iyer RK, Park H, Vunjak-Novakovic G (2008) Optical mapping of impulse propagation in engineered cardiac tissue. Tissue Eng Part A 15:851–860. 10.1089/ten.tea.2008.0223 PMC2759871 18847360

[CR34] Reed GW, Rossi JE, Cannon CP (2017) Acute myocardial infarction. Lancet 389(10065):197–210. 10.1016/S0140-6736(16)30677-8 27502078

[CR35] Rose RA, Jiang H, Wang XH, Helke S, Tsoporis JN, Gong N, Keating SCJ, Parker TG, Bacx P, Keating A (2007) Mesenchymal stromal cells acquire cardiac-specific markers but do not become functional cardiomyocytes and retain the stromal phenotype. Blood 110(11):1920. 10.1634/stemcells.2008-0329 18687994

[CR36] Rose RA, Jiang H, Wang X et al (2008) Bone marrow-derived mesenchymal stromal cells express cardiac-specific markers, retain the stromal phenotype, and do not become functional cardiomyocytes in vitro. Stem Cells 26(11):2884–2892. 10.1634/stemcells.2008-0329 18687994

[CR37] Scott L, Elídóttir K, Jeevaratnam K, Jurewicz I, Lewis R (2022) Electrical stimulation through conductive scaffolds for cardiomyocyte tissue engineering: Systematic review and narrative synthesis. Ann N Y Acad Sci 1515(1):105–119. 10.1111/nyas.14812 PMC9796457 35676231

[CR38] Scuteri A, Donzelli E, Foudah D, Caldara C, Redondo J, D’Amico G, Tredici G, Miloso M (2014) Mesengenic differentiation: Comparison of human and rat bone marrow mesenchymal stem cells. Int J Stem Cells 7(2):127–134. 10.15283/ijsc.2014.7.2.127 PMC4249895 25473450

[CR39] Shafy A, Lavergne T, Latremouille C, Cortes-Morichetti M, Carpentier A, Chachques JC (2009) Association of electrostimulation with cell transplantation in ischemic heart disease. J Thorac Cardiovasc Surg 138(4):971–978. 10.1016/j.jtcvs.2009.02.025 19660354

[CR40] Soma Y, Tani H, Morita-Umei Y, Kishino Y, Fukuda K, Tohyama S (2024) Pluripotent stem cell-based cardiac regenerative therapy for heart failure. Stem Cell Res 187:90–100. 10.1016/j.yjmcc.2023.12.001 38331557

[CR41] Steens J, Klein D (2018) Current strategies to generate human mesenchymal stem cells in vitro. Stem Cells Int Article 6726185. 10.1155/2018/6726185 PMC6129345 30224922

[CR42] Stoppel WL, Kaplan DL, Black LD III (2016) Electrical and mechanical stimulation of cardiac cells and tissue constructs. Adv Drug Deliv Rev 96:135–155. 10.1016/j.addr.2015.07.009 PMC4698182 26232525

[CR44] Tandon N, Goh B, Marsano A, Chao PG, Sorrentino CM, Gimble J, Vunjak-Novakovic G (2009) Alignment and elongation of human adipose-derived stem cells in response to direct-current electrical stimulation. Conf Proc IEEE Eng Med Biology Soc 6517–6521. 10.1109/IEMBS.2009.5333142 PMC2791914 19964171

[CR46] Tandon N, Marsano A, Maidhof R, Numata K, Montouri-Sorrentino C, Cannizzaro C, Voldman J, Vunjak-Novakovic G (2010) Surface-patterned electrode bioreactor for electrical stimulation. Lab Chip 10(6):692–700. 10.1039/b917743d PMC3618471 20221556

[CR45] Tandon N, Marsano A, Maidhof R et al (2011) Optimization of electrical stimulation parameters for cardiac tissue engineering. J Tissue Eng Regen Med 5(6):115–125. 10.1002/term.377 PMC3117092 21604379

[CR43] Tang M, Yang G, Jiang J, He X, Li H, Zhang M, Wu W, Liu X, Li L (2015) Expression of myocardial specificity markers MEF-2 C and Cx43 in rat bone marrow-derived mesenchymal stem cells induced by electrical stimulation in vitro. Sheng Wu Yi Xue Gong Cheng Xue Za Zhi 32(3):629–634 PMID: 26485990 26485990

[CR47] Thrivikraman G, Madras G, Basu B (2016) Electrically driven intracellular and extracellular nanomanipulators evoke neurogenic/cardiomyogenic differentiation in human mesenchymal stem cells. Biomaterials 77:26–43. 10.1016/j.biomaterials.2015.10.078 26576047

[CR48] Thrivikraman G, Boda SK, Basu B (2018) Unraveling the mechanistic effects of electric field stimulation towards directing stem cell fate and function: A tissue engineering perspective. Biomaterials 150:60–86. 10.1016/j.biomaterials.2017.10.003 29032331

[CR49] Tran L, Tam DNH, Elshafay A et al (2021) Quality assessment tools used in systematic reviews of in vitro studies: A systematic review. BMC Med Res Methodol 21(1):101. 10.1186/s12874-021-01295-w PMC8106836 33964880

[CR50] Vieira NM, Brandalise V, Zucconi E, Secco M, Strauss BE, Zatz M (2010) Isolation, Characterization, and Differentiation Potential of Canine Adipose-Derived Stem Cells. Cell Transplant 19(3):279–289. 10.3727/096368909X481764 19995482

[CR51] Wang B, Wang G, To F, Butler JR, Claude A, McLaughlin RM, Williams LN, de Jongh Curry AL, Liao J (2013) Myocardial scaffold-based cardiac tissue engineering: Application of coordinated mechanical and electrical stimulations. Langmuir 29:11109–11117. 10.1021/la401702w PMC3838927 23923967

[CR52] Wen L, Zhang C, Nong Y, Yao Q, Song Z (2013) Mild electrical pulse current stimulation upregulates S100A4 and promotes cardiogenesis in MSC and cardiac myocytes coculture monolayer. Cell Biochem Biophys 65(1):43–55. 10.1007/s12013-012-9402-x 22941361

[CR53] White SJ, Chong JJH (2020) Mesenchymal stem cells in cardiac repair: Effects on myocytes, vasculature, and fibroblasts. Clin Ther 42(10):1880–1891. 10.1016/j.clinthera.2020.08.010 32938532

[CR54] Yoshida S, Sumomozawa K, Nagamine K, Nishizawa M (2019) Hydrogel microchambers integrated with organic electrodes for efficient electrical stimulation of human iPSC-derived cardiomyocytes. Macromol Biosci 19:1970018. 10.1002/mabi.201900060 31038841

