# Supplementary material for: Effects of electrical stimulation on the cardiogenic differentiation of mesenchymal stem cells: A systematic review
Source: Cytotechnology. 2026 Oct 17;78(6):215. doi: 10.1007/s10616-026-01065-w (PMC13676602; doi:10.1007/s10616-026-01065-w)
Supplement: Supplementary file 1 [file 10616_2026_1065_MOESM1_ESM.pdf]

Online Resource 1. Complete search strategies for all databases

Article title: Effects of electrical stimulation on the cardiogenic differentiation of mesenchymal stem cells: A systematic review

Journal: Cytotechnology

Authors: Alejandra Sandoval Villarreal[1], Paola Andrea Neuta Arciniegas[1], John Jairo Cabrera López[2], Álvaro José Rojas Arciniegas[2]

Affiliations

[1] Research Group INBIOMIC, School of Bacteriology and Clinical Laboratory, Universidad del Valle.

[2] Faculty of Engineering and Basic Sciences, Universidad Autónoma de Occidente.

Corresponding author: Paola Andrea Neuta Arciniegas E-mail: paola.neuta@correounivalle.edu.co

Database	Search strategy	Search date	Records retrieved (n)
**Pubmed**	((("Mesenchymal Stem Cells"[Mesh]) AND ("Electric Stimulation"[Mesh] OR "Electric Stimulation Therapy"[Mesh])) AND (((("Myocytes, Cardiac"[Mesh]) OR "Connexin 43"[Mesh]) OR ("Troponin"[Mesh] OR "Troponin I"[Mesh])) OR ("Gap Junctions"[Mesh]))) OR (((¨mesenchymal stem cells¨ OR ¨Bone marrow mesenchymal stem cell¨) AND (¨Electric stimulation¨ OR ¨Electric Stimulation Therapy¨ OR ¨Electrical Stimulation¨)) AND (¨Cardiac Myocyte¨ OR ¨Cardiac Muscle Cell¨ OR ¨Cell Heart Muscle¨ OR ¨Connexin43¨ OR ¨Troponin¨ OR ¨Gap junctions¨))	July 2026	38
**Ovid**	(Mesenchymal Stem Cells or Bone marrow mesenchymal stem cell or mesenchymal stromal cell or MSC).mp. AND (Electric stimulation or Electric Stimulation Therapy or Electrical Stimulation).mp. AND (Myocytes, Cardiac or Cardiac Myocyte or Cardiac Muscle Cell or Cell Heart Muscle or Connexin43 or Troponin or Gap junctions).mp.	July 2026	17
**BVS**	células madre mesenquimatosas OR mesenchymal stem cells OR células-tronco mesenquimais OR cellules souches mésenchymateuses) AND (estimulación eléctrica OR electric stimulation OR electrical stimulation OR estimulação elétrica OR stimulation électrique) AND (miocardio OR myocardium OR miocárdio OR myocarde) AND instance:"regional"	July 2026	7

**EBSCO**	(Mesenchymal Stem Cells or Bone marrow mesenchymal stem cell or mesenchymal stromal cell or MSC) AND (Electric stimulation or Electric Stimulation Therapy or Electrical Stimulation) AND (Myocytes, Cardiac or Cardiac Myocyte or Cardiac Muscle Cell or Cell Heart Muscle)	July 2026	31
**ProQuest**	abstract(Mesenchymal stem cell OR Bone marrow mesenchymal stem cell) AND abstract(Electric stimulation OR Electric Stimulation Therapy) AND fulltext(Cardiac Myocyte OR Cardiac Muscle Cell OR Cell Heart Muscle OR Connexin43 OR Troponin OR Gap junctions)	July 2026	12
**Web of Science**	Mesenchymal Stem Cells OR mesenchymal stromal cell OR MSC (Abstract) AND Electric stimulation OR electrostimulation OR electrical stimulation (Abstract) AND Myocytes, Cardiac OR cardiomyocyte OR cardiac differentiation OR cardiogenic differentiation OR cardiomyogenic differentiation (All Fields)	July 2026	32
**Scopus**	(TITLE-ABS-KEY (Mesenchymal Stem Cells OR mesenchymal stromal cell OR MSC) AND ALL (Electric stimulation OR electrostimulation OR electrical stimulation) AND ALL (Myocytes Cardiac OR cardiomyocyte OR cardiac differentiation OR cardiogenic differentiation OR cardiomyogenic differentiation))	July 2026	23

Search strategies were updated in July 2026 and included a combination of controlled vocabulary terms (e.g., MeSH and DeCS, when available) and free-text keywords to improve search sensitivity and ensure comprehensive identification of potentially relevant studies. Search syntax and field tags were adapted according to the requirements of each database. The number of retrieved records corresponds to the results obtained prior to duplicate removal..mp. = multi-purpose search field, including title, abstract, subject heading words, keywords, and other indexed fields.
