# Supplementary material for: Effects of electrical stimulation on the cardiogenic differentiation of mesenchymal stem cells: A systematic review
Source: Cytotechnology. 2026 Oct 17;78(6):215. doi: 10.1007/s10616-026-01065-w (PMC13676602; doi:10.1007/s10616-026-01065-w)
Supplement: Supplementary file 3 [file 10616_2026_1065_MOESM3_ESM.pdf]

Online Source 3. Reasons for exclusion during title and abstract screening and full-text

Article title: Effects of electrical stimulation on the cardiogenic differentiation of mesenchymal stem cells: A systematic review

Journal: Cytotechnology

Authors: Alejandra Sandoval Villarreal[1], Paola Andrea Neuta Arciniegas[1], John Jairo Cabrera López[2], Álvaro José Rojas Arciniegas[2]

Affiliations

[1] Research Group INBIOMIC, School of Bacteriology and Clinical Laboratory, Universidad del Valle.

[2] Faculty of Engineering and Basic Sciences, Universidad Autónoma de Occidente.

Corresponding author: Paola Andrea Neuta Arciniegas E-mail: paola.neuta@correounivalle.edu.co

Screening stage	Reason for exclusion	Number of records (n)
**Title and abstract screening**	Studies not evaluating cardiogenic differentiation	27
	Non-primary publications (reviews and conference abstracts)	23
	Studies using additional cardiogenic induction strategies	11
	Non-MSC studies	6
	No electrical stimulation intervention	5
**Reports not retrieved**		3
**Full text assessment**	Genetic induction approaches	3
	Use of 5-azacytidine	1
	In vivo electrical stimulation study	1
**Total records excluded**		**80**
